# Single-FiO_2_ lung modelling with machine learning: a computer simulation incorporating volumetric capnography

**DOI:** 10.1007/s10877-023-00996-5

**Published:** 2023-04-01

**Authors:** Thomas J. Morgan, Peter H. Scott, Adrian N. Langley, Robin D. C. Barrett, Christopher M. Anstey

**Affiliations:** 1 Mater Research and University of Queensland, Stanley Street, South Brisbane, Brisbane, QLD 4101 Australia; 2 Intensive Care Department, Mater Health Services, Stanley Street, South Brisbane, Brisbane, QLD 4101 Australia; 3 University of Queensland, Brisbane, QLD 4072 Australia; 4 Freelance Computer Programmer, Brisbane, QLD Australia; 5 Griffith University, Gold Coast, QLD 4215 Australia

**Keywords:** Deep learning, Low V/Q, Machine learning, MIGET, Shunt, Simulation, Single—FiO_2_

## Abstract

We investigated whether machine learning (ML) analysis of ICU monitoring data incorporating volumetric capnography measurements of mean alveolar PCO_2_ can partition venous admixture (VenAd) into its shunt and low V/Q components without manipulating the inspired oxygen fraction (FiO_2_). From a 21-compartment ventilation / perfusion (V/Q) model of pulmonary blood flow we generated blood gas and mean alveolar PCO_2_ data in simulated scenarios with shunt values from 7.3% to 36.5% and a range of FiO_2_ settings, indirect calorimetry and cardiac output measurements and acid- base and hemoglobin oxygen affinity conditions. A ‘deep learning’ ML application, trained and validated solely on single FiO_2_ bedside monitoring data from 14,736 scenarios, then recovered shunt values in 500 test scenarios with true shunt values ‘held back’. ML shunt estimates versus true values (n = 500) produced a linear regression model with slope = 0.987, intercept = -0.001 and R^2^ = 0.999. Kernel density estimate and error plots confirmed close agreement. With corresponding VenAd values calculated from the same bedside data, low V/Q flow can be reported as VenAd—shunt. ML analysis of blood gas, indirect calorimetry, volumetric capnography and cardiac output measurements can quantify pulmonary oxygenation deficits as percentage shunt flow (V/Q = 0) versus percentage low V/Q flow (V/Q > 0). High fidelity reports are possible from analysis of data collected solely at the operating FiO_2_.

**Supplementary Information:**

The online version contains supplementary material available at 10.1007/s10877-023-00996-5.

## Introduction

We have previously described quantification of pulmonary gas exchange from machine learning (ML) analysis of bedside monitoring data [1]. In that study we showed by computer simulation that trained ML analysis of blood gas, indirect calorimetry, and cardiac output measurements can generate gas exchange assessments based on an adaptation of West’s ventilation/perfusion (V/Q) lung model [2, 3]. The assessments resemble multiple inert gas technique (MIGET) reports, albeit free of MIGET’s technical challenges [4, 5].

The reference methodology in this ‘scaled back’ MIGET space is the Automatic Lung Parameter Estimator (ALPE) [6–9], which analyses a similar suite of inputs. Both methods address a major limitation of the three-compartment lung model of Riley and Cournand [10, 11], which groups all oxygen transfer deficits within its shunt compartment and reports them as ‘venous admixture’ (VenAd). As highlighted previously [1], the ability to distinguish the relative influences of true shunt versus low V/Q effects in hypoxemia can influence management in conditions such as COVID-19 pneumonia [12].

Our ‘adapted West’ model enables separate quantification of these components by partitioning VenAd into ‘shunt’ and ‘low V/Q’, where low V/Q = VenAd—shunt. Both indices are expressed as percentages of pulmonary blood flow. ALPE quantifies low V/Q by a separate metric, as a notional PO_2_ gradient between alveolar gas and pulmonary end-capillary blood [9].

A drawback common to both methods is the need to collect data at more than one inspired oxygen fraction (FiO_2_), introducing potential signal distortion from absorption atelectasis and altered hypoxic pulmonary vasoconstriction [13, 14]. For high fidelity estimates the adapted West method currently requires blood gases measured at two structured FiO_2_ settings [1], whereas ALPE institutes as many as four FiO_2_ shifts for an extended series of pulse oximetry measurements [8].

It has become apparent that the ‘Two–FiO_2_’ requirement of our approach could be eliminated by incorporating mean alveolar PCO_2_ (mean PACO_2_), measurable at the bedside using volumetric capnography [15]. With these additional monitoring data, just one set of measurements collected at any operating FiO_2_ should suffice. If shown to be accurate and reproducible, this ‘Single–FiO_2_’ method would enable critical care practitioners to distinguish shunt from low V/Q contributions to VenAd rapidly without altering the operating FiO_2_.

We therefore tested the following hypotheses in silico:

1. Data from blood gas analysis, indirect calorimetry, cardiac output, and volumetric capnography measurements analyzed in combination can quantify oxygenation deficits in terms of percentage shunt flow (V/Q = 0) versus percentage low V/Q flow (V/Q > 0).
2. Consistent reports can be generated by ML analysis of data collected solely at the operating FiO_2_.

## Materials and methods

We tested the above hypotheses through a ‘reverse engineering’ simulation.

**Fig. 1:**
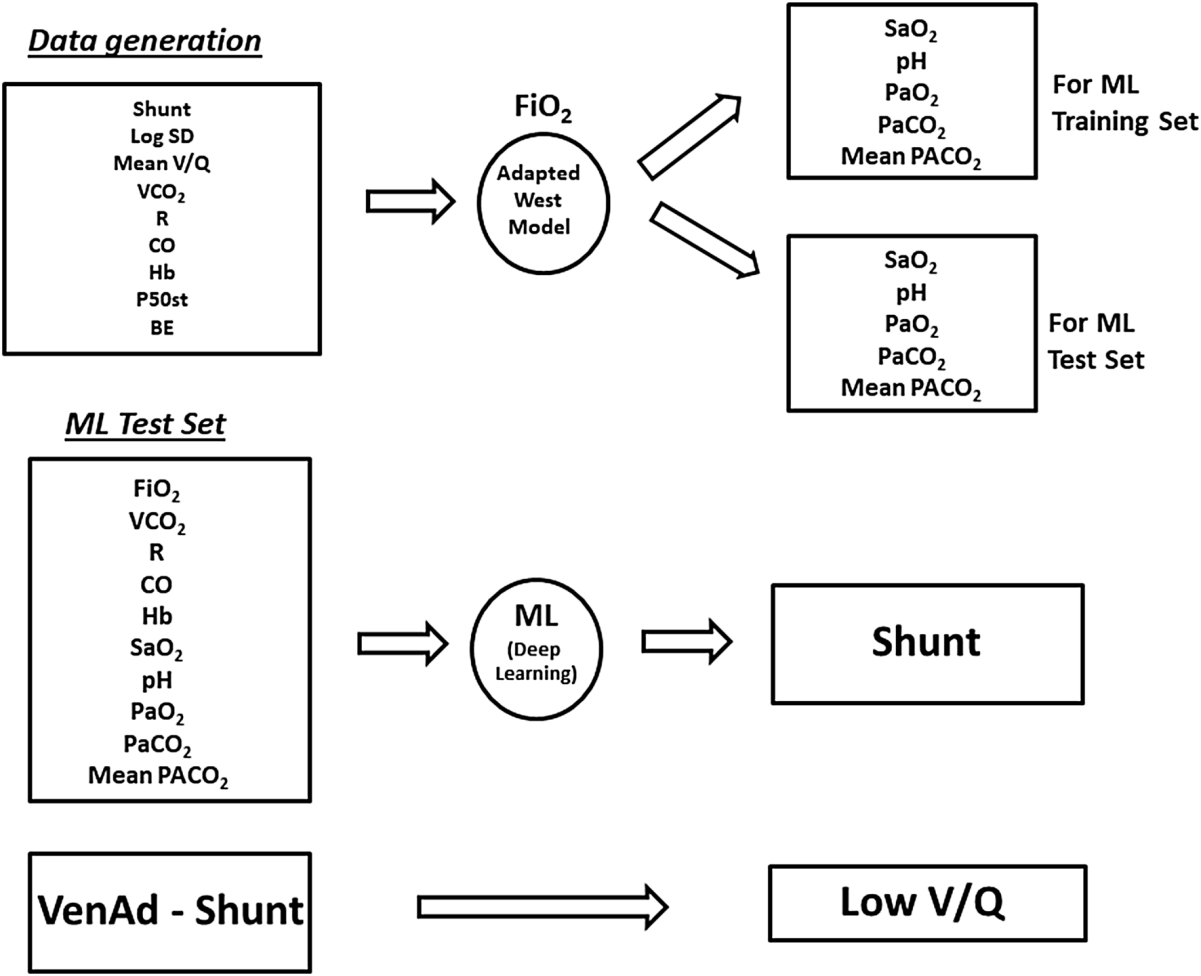
Architecture of the ‘reverse engineering’ method. ‘ML’ is Machine Learning. ‘Venad’ is venous admixture, calculated as per Eq. 10 in Supplementary Material. Other abbreviations as in Tables 1 and 2

The simulation in brief (Fig. 1).

1. Using the adapted West lung model, we generated blood gas and mean PACO_2_ data at various FiO_2_ settings from simulated indirect calorimetry and cardiac output measurements across a range of shunt values, arterial hemoglobin—oxygen saturations (SaO_2_), and acid- base and hemoglobin oxygen affinity conditions.
2. We divided these data into a training set and a test set.
3. With the training set we programmed a ML application to recover shunt values solely from Single-FiO_2_ ‘bedside’ monitoring data.
4. We then tasked the trained ML application with the ‘blinded’ recovery of the test set shunt values.
5. Precise recovery of shunt values using only ‘bedside’ data would document the accuracy and reproducibility necessary for clinicians to determine shunt contributions to VenAd.
6. Low V/Q contributions could then be calculated as ‘VenAd—shunt’.

### The lung model

The ‘adapted West’ model currently runs via VBA sub-routines on Excel (Microsoft, Redmond, WA). Now updated to include N_2_ exchange, it incorporates log normal distributions of pulmonary blood flow across 20 compartments spanning a broad range of V/Q ratios, plus a separate shunt compartment (V/Q = 0). A model description including core equations is available in the Supplementary Material.

### The simulation in more detail

More than 15,200 unique combinations of model inputs within pre-defined ranges (Table 1) were created by a Python program. For each combination the adapted West model generated blood gases and mean PACO_2_ values with the FiO_2_ adjusted for SaO_2_ ≥ 0.87 ≤ 0.98 (Table 2). As with our previous ‘Two-FiO_2_’ method evaluation [1], simulated scenarios encompassed a cross section of O_2_ consumption (VO_2_) and delivery, CO_2_ production (VCO_2_) and transport, hemoglobin-oxygen affinity, and respiratory and metabolic acid–base status.

**Table 1:** Monitoring inputs used by the model to generate the ranges of scenario blood gases and PACO_2_ values in Table 2

Variable	Range
Shunt (% of pulmonary blood flow)	7.3–36.5
Log SD	0.36–1.83
Mean V/Q	0.21–1.25
VCO_2_ (mL/min)	185–223
R	0.57–1.05
Hemoglobin (G/dL)	6.0–17.3
P50st (mm Hg)	20.8–31.2
Base Excess (mEq/L)	− 6.5–4.9
CO (L/min)	4.32–6.28

V = volume of inspired gas. Q = volume of mixed venous blood. SD = standard deviation VCO_2_ = Total carbon dioxide production rate; R = respiratory exchange ratio (VCO_2_/VO_2_); P50st = standard P50; CO = cardiac output

**Table 2:** Model-generated blood gas and mean PACO_2_ data

Variable	Range
FiO_2_	0.21–0.99
SaO_2_	0.87–0.98
pH	7.02–7.56
PaO_2_ (mm Hg)	41.8–127.7
PaCO_2_ (mm Hg)	23.8–111.6
Mean PACO_2_ (mm Hg)	14.2–77.1

FiO_2_ = inspired oxygen concentration; SaO_2_ = arterial oxygen saturation; PaO_2_ = oxygen partial pressure in arterial blood; PaCO_2_ = carbon dioxide partial pressure in arterial blood; Mean PACO_2_ = mean carbon dioxide partial pressure in alveolar gas

The final dataset was subjected to simple randomization, then divided into a training set of 14,736 data—rows and a test set of 500 data—rows. We settled for 500 rows as the final test set rather than 10% of the total dataset (approximately 1500 rows) since these were sufficient for meaningful statistical analysis. Following ML training and validation (see below), shunt values were recovered for each test scenario (n = 500) with the true shunt values ‘held back’.

Recovery was performed solely by ML analysis of the following ten ‘bedside’ data elements: FiO_2_, blood hemoglobin concentration (Hb), SaO_2_, arterial pH, arterial oxygen tension (PaO_2_) arterial CO_2_ tension (PaCO_2_), mean PACO_2_, cardiac output, VCO_2_ and R (the respiratory exchange ratio VCO_2_/VO_2_). Recovered shunt estimates were then compared with their ‘true’ shunt counterparts.

### ML training

[See ‘Machine (Deep) Learning terminology’ and ‘Deep Learning Model—more detail’ in Supplementary Material].

The ‘Keras’ library [16, 17] was used to construct deep learning models, each with an input layer of 10 features, plus densely connected intermediate layers and a final layer with one unit. The ReLU activation function was utilised. The optimizer was RMSprop, and the loss function was MSE (mean squared error).

The dataset was shuffled, and input features normalized. Overfitting could not be induced with increased numbers of layers, units/layer, and training epochs, consistent with a simulated dataset free from observational errors and from the inherent variation of natural phenomena.

We selected a model with six densely connected intermediate layers and 128 units/layer, resulting in 84,097 trainable parameters. The model was trained to predict shunt using 500 epochs of a dataset of 14,736 physiological records.

### Sensitivity analyses

We assumed FiO_2_ accuracy and determined sensitivities to the remaining nine ML inputs. A representative scenario was selected in which both shunt and low V/Q blood flow contribute to a significant gas exchange deficit, as summarized by the VenAd value. The nine monitoring inputs could then be varied individually above and below their ‘true’ values while effects on VA and shunt (and therefore low V/Q, defined as VenAd—shunt) were tracked in graphic format.

This exercise was conducted using a direct (non-ML) method of model back-calculation (described in the Supplementary Material). Corresponding VenAd calculations to accompany shunt estimates were performed by application of Eq. 10 in the Supplementary Material.

### Statistical methods

The data set consisted of binary pairs of actual and estimated data for shunt. To demonstrate the fit of the model, univariate regression was undertaken to assess the relationship between each estimated result and its actual partner, using the actual partner as the dependent variable. Results were reported as the regression slope, regression constant and the coefficient of determination, the R^2^ value. With perfect agreement between actual and estimated values, these parameters would be 1.00, 0.00 and 1.00 respectively. The 95% confidence interval and associated p-value were also reported.

Deviations of estimated from actual values were calculated and plotted as ‘estimate – actual’. Mean (SD) and median (IQR) were summarized with the data range.

A kernel density estimate (KDE) plot was also constructed. This enabled a visual representation of both the underlying distribution of the data and the accuracy of the fit of the predicted shunt values.

The level of significance was set at α < 0.05 for all relevant analyses. The statistical analysis and associated plots were undertaken using STATA™ (ver 17.0).

## Results

**Fig. 2:**
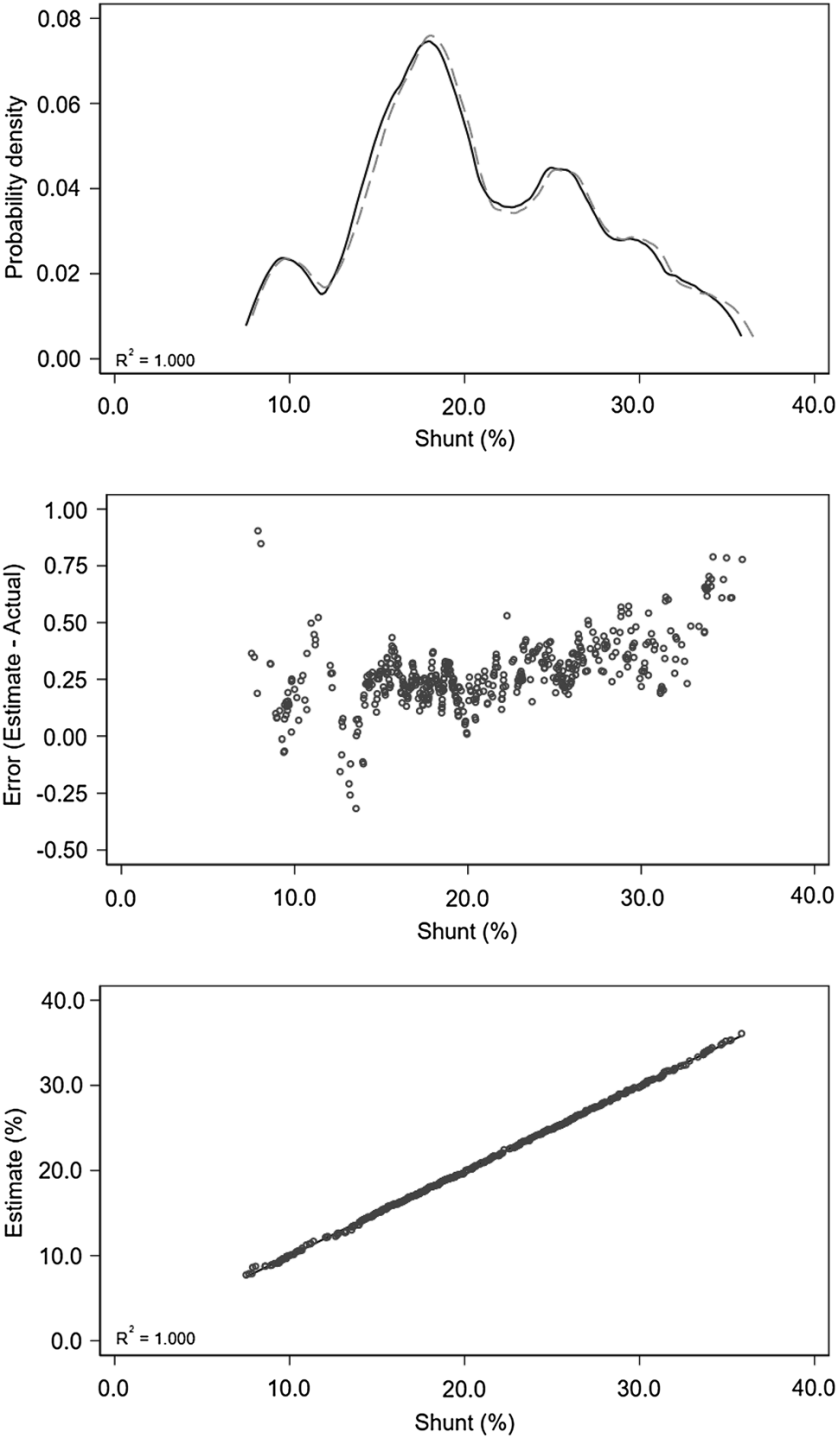
Shunt estimates versus true values. Three subplots share the same X-axis scale. The kernel density estimate (KDE) plot (upper graph) illustrates the distribution of observations for the independent variable along with goodness of fit. The Y-axis in the KDE plot is dimensionless. The solid line (true shunt values) and the dashed line (shunt estimates) are closely aligned. Close agreement, slightly reduced at Shunt ≤ 15%, is evident in the error plot (middle graph), and in the plot of true shunt versus shunt estimates (lower graph)

Linear regression and error calculation results are set out in Tables 3 and 4, while relationships between true and estimated shunt values are illustrated graphically in Fig. 2 by KDE and error plots, along with a plot of true shunt versus estimates.

**Table 3:** Univariate linear regression for shunt. Sample size was 500

Variable	β1	95% CI	p value	β0	R^2^
Shunt	+ 0.987	+ 0.985, + 0.988	< 0.001	− 0.001	0.999

β1 = regression coefficient. β0 = regression equation intercept. R^2^ = coefficient of determination

**Table 4:** Error calculation (actual – estimate)

Variable	Mean (SD)	Median (IQR)	Range
Shunt	+ 0.276 (0.148)	+ 0.260 (+ 0.200, + 0.343)	− 0.316 to + 0.905

Close agreement is demonstrated. The calculated error although small, is slightly right skewed (Skewness =  + 0.53) and not normally distributed (Shapiro–Wilk p < 0.001), with most occurring at Shunt < 15%. The plot of actual shunt versus shunt estimates confirms overall close agreement, while best approximating the line of identity when Shunt ≥ 15% (Fig. 2). These findings are also reflected in the adjacent error plot.

### Sensitivity analysis

The nine model inputs selected for the sensitivity evaluation were:

Hb 9.30 g/dL, pH 7.364, PaCO_2_ 39.4 mm Hg, PaO_2_ 69.0 mm Hg, SaO_2_ 0.94,mean PACO_2_ 30.1 mm Hg, cardiac output 5.25 L/min, VCO_2_ 187 mL/min, R 0.74.

At FiO_2_ = 0.38, model calculations produced the following estimates which determine the shunt / low V/Q split:

VenAd 18.5%

Shunt 10.8%

**Fig. 3:**
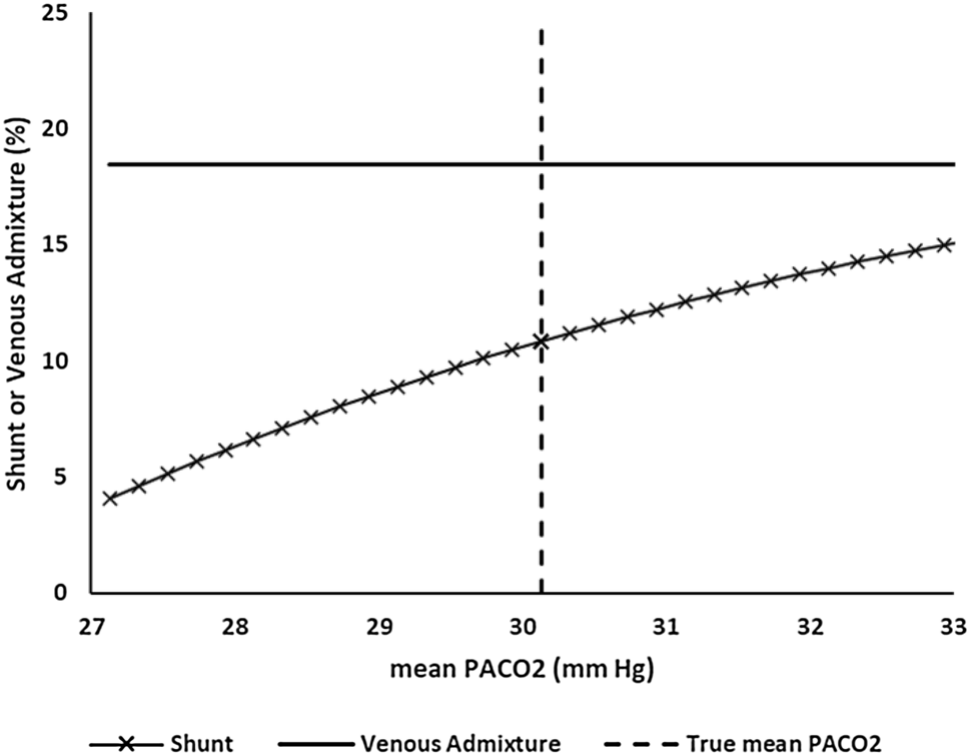
Variation of mean PACO_2_ (mean alveolar PCO_2_) measurements above and below the ‘true’ value with corresponding venous admixture and shunt percentages

**Fig. 4:**
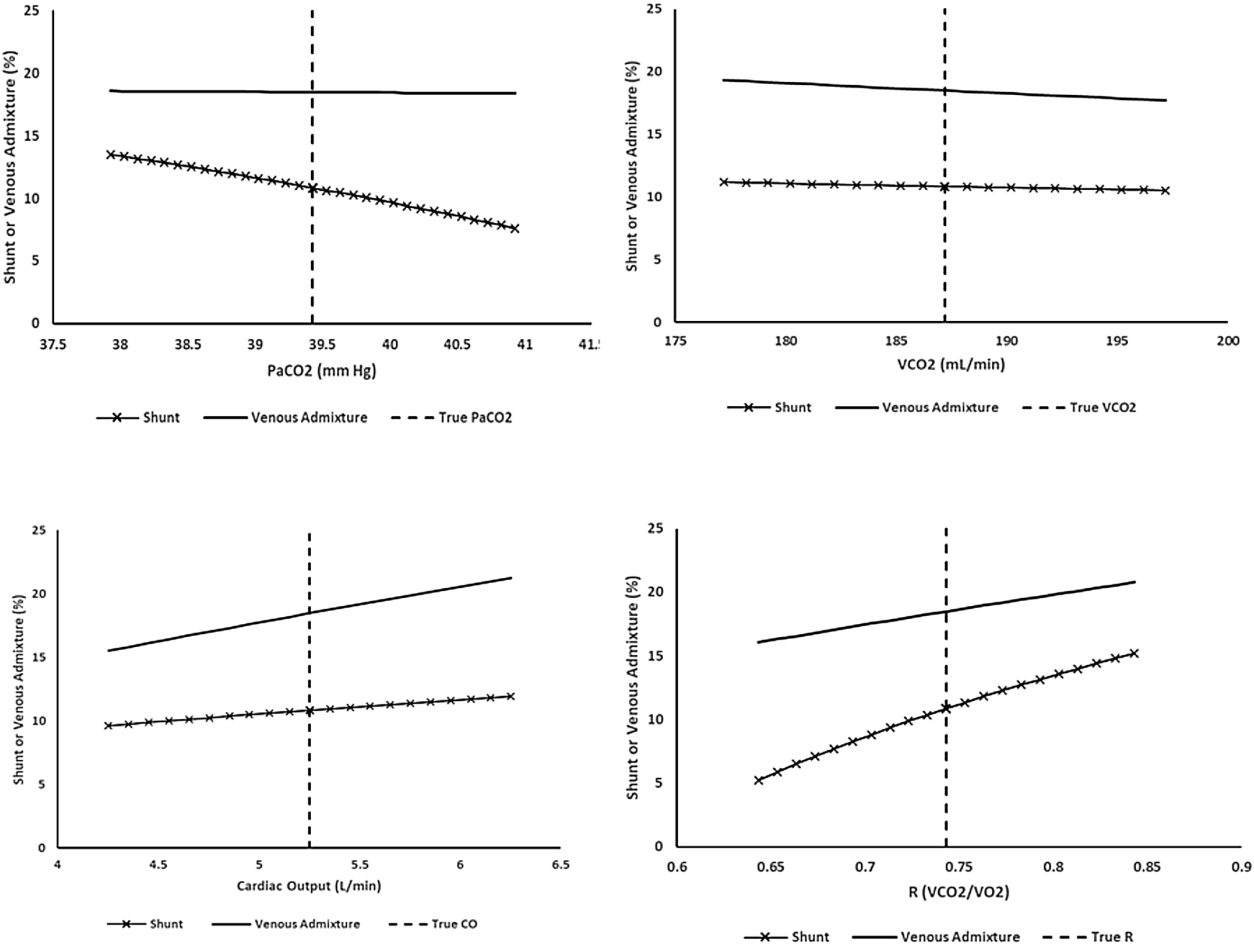
Variation of PaCO_2_, VCO_2_, cardiac output and R (respiratory exchange ratio) values above and below ‘true’ values with corresponding venous admixture and shunt percentages

**Fig. 5:**
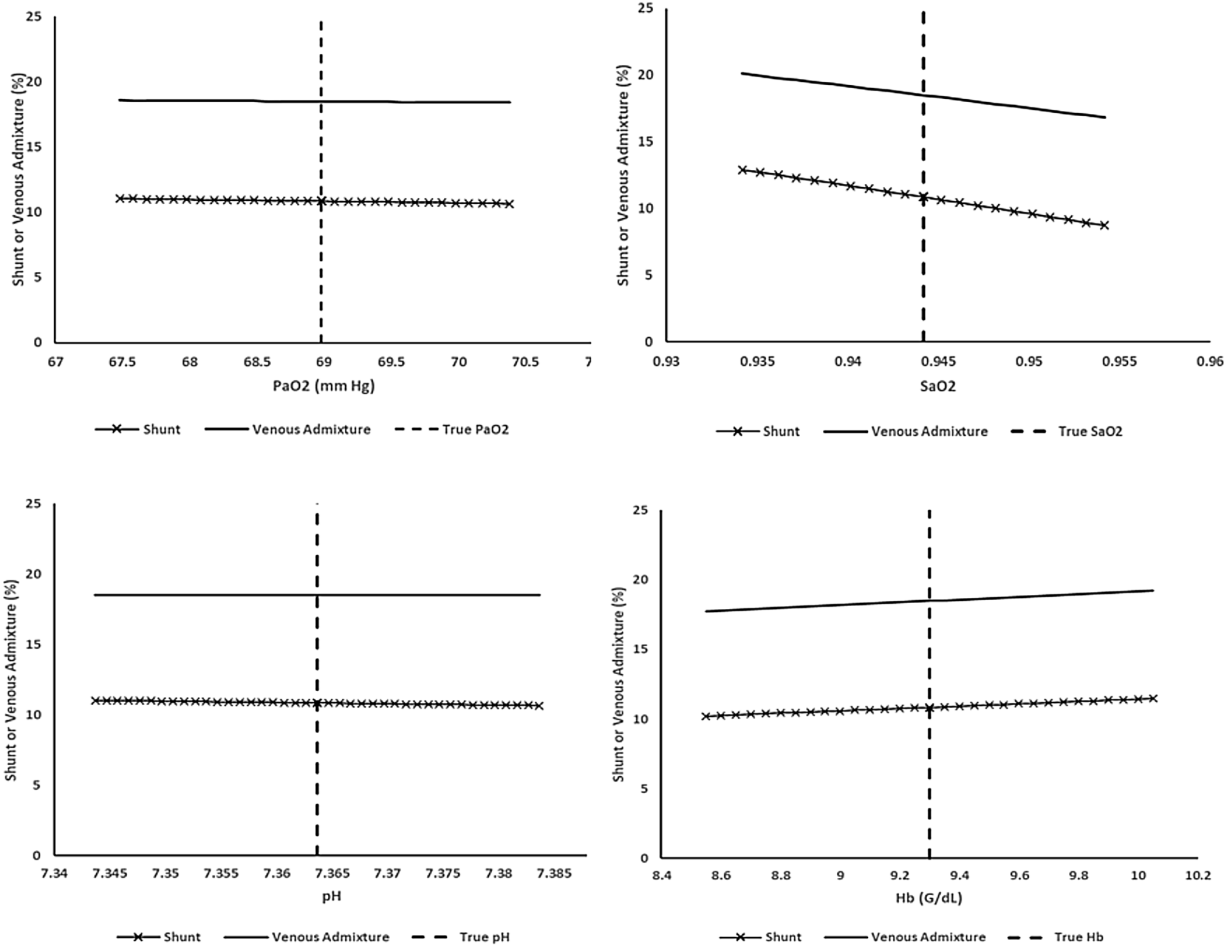
Variation of PaO_2_, SaO_2_, pH and Hb above and below the ‘true’ values with corresponding effects on venous admixture and shunt percentages

Responses to individual input variations are illustrated in Figs. 3, 4, 5. PaO_2_ and pH variation had the smallest overall effects on shunt percentage and VenAd. Each of the remaining monitoring inputs produced distinct shifts in shunt percentage. Concurrent alterations in VenAd were also evident, except with variations in PaCO_2_ and mean PACO_2_.

## Discussion

In a computer simulation we recovered individual shunt values from 500 diverse gas exchange scenarios using a ‘Deep Learning’ ML analysis of monitoring data. The input data consisted of the operating FiO_2_ plus nine ‘bedside’ monitoring measurements including blood gas and mean PACO_2_ values created from a 21- compartment model of pulmonary blood flow. Collected without FiO_2_ manipulation, these were sufficient for accurate shunt recovery. Therefore unlike the ALPE method [8, 9] or our Two-FiO_2_ method [1], it should be possible to dispense with FiO_2_ ‘switching’ using this approach. Since VenAd values are calculated directly from the same data, corresponding ‘low V/Q’ components can be reported simultaneously as ‘VenAd—shunt’.

The small error increase observed at shunt values ≤ 15% is possibly a reflection of abbreviated ML training in lower shunt scenarios. Reduced scenario numbers in this region (although also at shunt values ≥ 30%) are evident in the test set KDE plot (Fig. 2). Scenario distributions in the training set are similar (plot not shown).

As with the Two-FiO_2_ method [1], the requisite data elements for ML analysis were sourced from blood gas, indirect calorimetry, and cardiac output measurements, but with the addition of mean PACO_2_ measurements by volumetric capnography. Corresponding mean PACO_2_ values could be calculated from the model with relatively minor adjustments. This factor prompted our selection of mean PACO_2_ over end-tidal PCO_2_, notwithstanding the promise shown by the latter measurement in severity stratification of acute respiratory distress syndrome (ARDS) [18].

Single–FiO_2_ quantification of gas transfer, in other words quantification without FiO_2_ manipulation, can be performed rapidly at the operating FiO_2_. There are no re-equilibration intervals, and there should be no possibility of signal distortion from absorption atelectasis [13] and altered hypoxic pulmonary vasoconstriction [14]. However, Single—FiO_2_ indices in current use have significant drawbacks. The difficulty with the three-compartment lung model of Riley and Cournand [10, 11] was canvassed in the Introduction, while simpler Single—FiO_2_ indices such as the A-a gradient and the PaO_2_/ FiO_2_ (PF) ratio are inconsistent instruments [19] providing limited information on the underlying lung pathophysiology.

‘Scaled back MIGET’ approaches such as ALPE [8, 9] and our proposed ‘adapted West’ model [1] can provide physiological detail to assist physician decision making, the focus in this case being the separate quantification of shunt and low V/Q effects [8, 12]. More generally they represent an opportunity to improve severity and prognostic stratifications, for example in ARDS [20, 21]. To date their main downside has been a requirement for Two-FiO_2_ [1] or even Multi–FiO_2_ [8, 9] data input.

We have shown in this ‘reverse engineering’ simulation that the adapted West model can function using ML at a high level of accuracy in Single–FiO_2_ mode provided mean PACO_2_ values are included in the data mix. With the advent of volumetric capnography [15], this can be accomplished without collection of expired gas in Douglas bags. The only additional requirement was incorporation of N_2_ exchange in the model to enable more accurate estimates of mean PACO_2_.

Having both shunt and low V/Q estimates expressed as percentages of pulmonary blood flow can simplify interpretation of lung pathophysiology and thereby facilitate management decisions. As discussed previously [1], a severely reduced PF ratio in a patient with ARDS typically flags extensive lung consolidation causing a large right to left shunt. Under these circumstances practitioners may initiate recruitment maneuvers, after which positive end expiratory pressure (PEEP) settings are commonly reset to maintain recruited lung regions [19].

However, identical oxygenation disturbances can occur despite well—aerated lung with minimal true shunt. For example, in COVID -19 pneumonia there may be extensive pulmonary micro—thromboses diverting mixed venous blood flow through low V/Q compartments [12, 22]. In this latter scenario recruitment maneuvers and significant PEEP manipulations are likely to be counterproductive, the priority now being limitation of lung over-distension. Based on our findings, rapid distinction between these two scenarios should be possible by ML modelling of pulmonary blood flow distribution from data collected solely at the operating FiO_2_.

When upgrading from Two-FiO_2_ to Single-FiO_2_ mode, volumetric capnography could simply be added to the existing input mix. Alternatively, since VCO_2_ measurements would then be available from two devices, volumetric capnography could replace indirect calorimetry provided an appropriate respiratory exchange ratio (R = VCO_2_/VO_2_) was also assigned. For example, R = 0.90 was the apparent approximate mean R value in a recent report of patients with ARDS [8]. However, incorrect R values reduce accuracy (Fig. 4).

Sensitivity analyses raise other caveats. In Figs. 3, 4, 5 estimated shunt / low V/Q splits are shown to be variously sensitive to the nine bedside input values. In some cases, the VenAd calculation (which then defines low V/Q as ‘VenAd—shunt’) is also altered. Of note, mean PACO_2_ variability can cause significant shifts. Inspection of Fig. 3 shows that a 1 mm Hg variation above or below the ‘true’ mean PACO_2_ value moved the estimated shunt by approximately 2% of pulmonary blood flow up or down respectively. Sensitivity may vary in other gas exchange scenarios. For example, we observed during the evaluation that mean PACO_2_ sensitivity is reduced when true shunt dominates the VenAd, whereas it can exceed 3% / mm Hg when low VQ flow is the primary gas exchange abnormality.

To report mean PACO_2_ the volumetric capnograph scans single breath waveforms to determine the geometric mid-point of Phase 3 [23]. Precise identification of Phase 3 commencement is therefore key, but can be uncertain when there are waveform abnormalities [24]. Single breath waveforms are distorted by several factors known to impact end-tidal PCO_2_ measurement, which include tachypnea, bronchospasm, bronchial intubation, partial airway obstruction, and ventilator ‘malfunctioning’ [24].

Considering that there are another eight inputs in addition to mean PACO_2_, each with measurement and biological variation, the Single—FiO_2_ method if introduced clinically would ideally incorporate multiple data sampling across a rolling time segment, for example the prior five minutes. It would necessitate capturing mean PACO_2_ values breath—to—breath, along with streamed inputs of blood gases, cardiac output, VO_2_ and VCO_2_. Continuous shunt/low V/Q updates could then be produced in close to real time with relatively minor damping.

However the only continuous blood gas device in current use is confined to pump flow measurements during cardiopulmonary bypass [25]. Continuous optode-based analyzers are no longer available for general clinical use mainly due to problems with measurement artifact [26]. That being so, snapshot blood gases with the rest in rolling format would be ‘the next best thing’. Even with a complete downgrade to snapshot inputs across the board, shunt values could be estimated by ML using both Single-FiO_2_ and Two-FiO_2_ methods [1], affording practitioners three contemporaneous shunt/low V/Q split estimates by two alternative methods.

## Conclusion

We conclude based on computer simulations of diverse gas exchange scenarios that data collected at a single FiO_2_ from blood gas, indirect calorimetry, cardiac output, and volumetric capnography measurements can be used to quantify pulmonary oxygenation deficits as percentage shunt flow (V/Q = 0) versus percentage low V/Q flow (V/Q > 0). ML analysis, a game-changer for the Two-FiO_2_ Adapted West method [1], is now shown to perform with similar accuracy in Single-FiO_2_ mode, so that rapid shunt/low VQ estimates can be performed at the operating FiO_2_ without impacting gas transfer.

## Supplementary Information

Below is the link to the electronic supplementary material.

Supplementary file1 (DOCX 434 KB)
