# Supplementary material for: Single-FiO2 lung modelling with machine learning: a computer simulation incorporating volumetric capnography
Source: J Clin Monit Comput. 2023 Apr 1;37(5):1303–11. doi: 10.1007/s10877-023-00996-5 (PMC10066977; doi:10.1007/s10877-023-00996-5)
Supplement: Supplementary file 1 — Supplementary file1 (DOCX 434 KB) [file 10877_2023_996_MOESM1_ESM.docx]

Supplementary Material

The ‘Adapted West’ lung model

The ‘Adapted West’ model of pulmonary gas exchange is based on the approach of West [1] with certain modifications. It was developed in Excel (Microsoft, Redmond, WA) and is currently run on that platform using VBA sub-routines. A Python version and an App are under development.

The model can be configured to generate arterial blood gases (including hemoglobin oxygen saturation) and mean alveolar PCO_2_ values (mean PACO_2_) at any inspired oxygen concentration (FiO_2_) according to nine constraints. These consist of the three governing parameters of pulmonary blood flow distribution (shunt, log SD and mean), and six selected monitoring inputs (Table 1, main article).

The following are key model characteristics:

1. One shunt compartment (V/Q = 0) and 20 gas exchanging compartments.
2. Modelling centered on pulmonary blood flow distribution.
3. Non-shunt pulmonary blood flow distributed on a log normal basis across a spread of compartmental V/Q ratios as governed by mean V/Q and log standard deviation (log SD).
4. Pulmonary blood flow distribution is thus defined in full by three parameters: shunt, log SD and mean.
5. Compartmental ventilation is a dependent variable derived from compartmental blood flow and its allocated V/Q ratio. The distribution of V versus V/Q ratios across the 20 ventilated compartments is log normal, with the same log SD as for Q but with a separate mean which is defined by blood flow parameters [Figures 1 (s) and 2 (s)].
6. In healthy lungs blood flow and ventilation have narrow distributions with means centred near V/Q = 1 [(Figure 1(s)].
7. As log SD increases, V/Q heterogeneity expands while the two means diverge [Figure 2(s)]. A greater proportion of mixed venous blood then equilibrates in low V/Q compartments reducing pulmonary capillary oxygen uptake, culminating in zero uptake in pure shunt (V/Q = 0).
8. At the same time a greater proportion of alveolar ventilation is directed to higher V/Q compartments, reducing the efficiency of CO_2_ elimination.


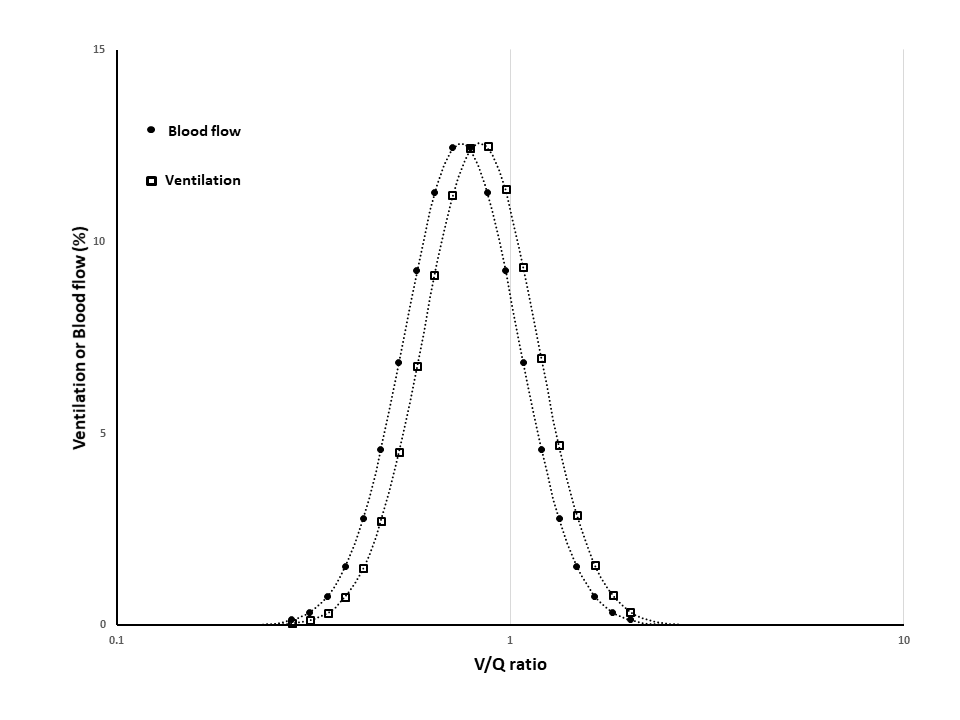


Figure 1 (s). Blood flow and corresponding ventilation in healthy lungs according to V/Q ratios in 20 gas exchanging compartments. Blood flow parameters: Shunt 0%, log SD = 0.33, V/Q distributional mean = 0.75. Ventilation parameters: log SD = 0.33, V/Q distributional mean = 0.84. In healthy lungs blood flow and ventilation distributions are narrow and close to unimodal.


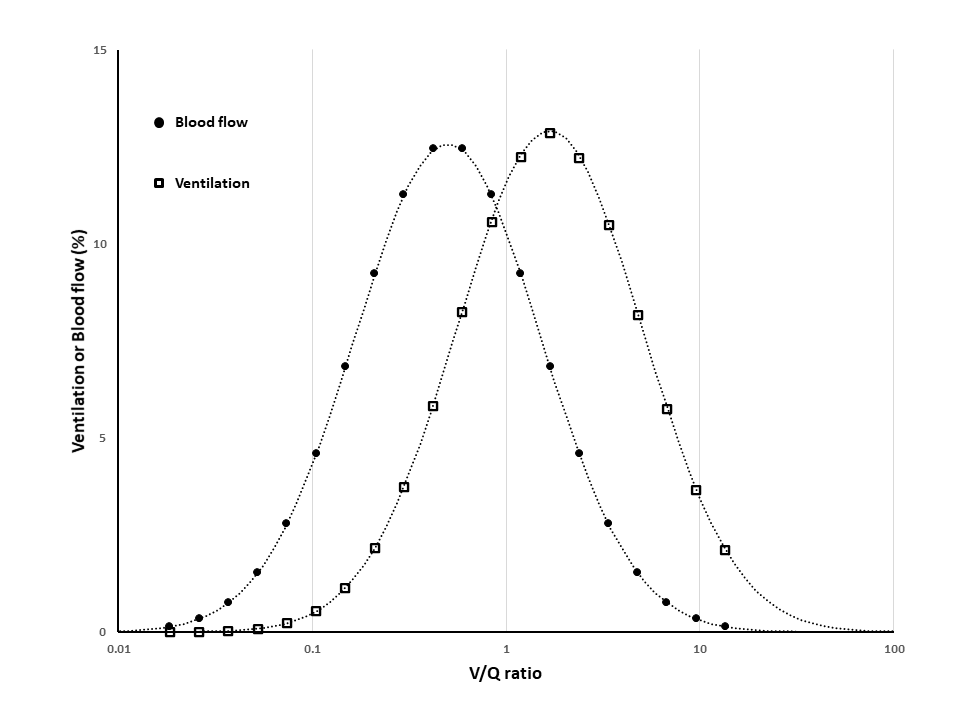


Figure 2 (s). Blood flow and corresponding ventilation in lungs with significant V/Q mismatch. Blood flow parameters: Shunt 0%, log SD = 1.1, V/Q distributional mean = 0.5. Ventilation parameters: log SD = 1.1, V/Q distributional mean = 1.65. Note the diverging means with the increase in V/Q heterogeneity.

More detail

1. N_2_ exchange is now incorporated.
2. The inclusion of N_2_ exchange can further reduce expired gas volumes, especially at higher FiO_2_ settings in compartments with low V/Q values [1]. The model is programmed to treat calculated expired volumes ≤ 0 as compartmental collapse from loss of N_2_ splinting. Any ‘collapsed’ compartment is then switched to shunt mode, with zero expired gas and with capillary output unaltered as mixed venous blood.
3. Blood CO_2_ content (CbCO_2_) is calculated by applying the Douglas equation [2] (Core Equations 2 and 3) rather than via the Kelman sub-routine used by West [3].The Douglas equation is a 1988 update of the ‘McHardy – Visser’ equation [4], with new constants derived from experiments on volunteers [2]. Agreement with these experimental data was closer than with the relevant Kelman sub-routine [3].
4. Hemoglobin – oxygen dissociation is modelled using a version of Siggaard-Andersen’s tan H equation [5] (Core Equation 8), again in preference to the earlier Kelman sub-routine [6]. The tan H method is well-established and maintains close agreement with the standard curve if SO_2_ ≤ 0.97.
5. The model applies a fixed BE Haldane coefficient of 0.22 [7] (Core Equation 9) to express saturation - induced changes in metabolic acid-base status and thus total [CO_2_].
6. As stated, compartmental ventilation is a dependent variable defined by compartmental blood flow and its allocated V/Q ratio. In the original West model distributions of blood flow and ventilation are imposed separately as independent variables (and also assessed separately by MIGET), with compartmental V/Q ratios themselves becoming dependent variables [1].
7. There are 20 gas exchanging compartments plus shunt in the Adapted West model, whereas MIGET normally has 50 gas exchanging compartments. According to West, increasing model compartment numbers above 10 has minimal effect on VO_2_, VCO_2_, PaO_2_ and PaCO_2_ [8].
8. Mean PACO_2_ is now reported (Equation 37) to facilitate bedside shunt calculation.

Core Equations

1. CbO_2_ (Blood oxygen content in ml/dL)

$$CbO_{2}=1.34.Hb\times S+0.003\times PO_{2}$$

Hb = Blood hemoglobin concentration (g/dL)

S = fractional saturation of hemoglobin with oxygen

PO_2_ = partial pressure of oxygen in blood (mm Hg)

1. CpCO_2_ (Plasma CO_2_ content in ml/dL)

$$CpCO_{2}=2.226\times0.03.PCO_{2}.\left( 1+{10}^{pH-6.1} \right)$$

PCO_2_ = partial pressure of carbon dioxide in blood (mm Hg)

1. CbCO_2_ (Blood CO_2_ content in ml/dL)

$$CbCO_{2}=CpCO_{2}\times\left[ 1-\frac{0.0289\times Hb}{\left( 3.352-0.456\times S \right).\left( 8.142-pH \right)} \right]$$

1. Bicarb (Plasma bicarbonate in mmol/L)

$$Bicarb=0.03\times PCO_{2}\times{10}^{pH-6.1}$$

2. BE (Base excess in mEql/L) [9]

$$BE=\left( 1-0.0143\times Hb \right)\times\left[ Bicarb-24.4+\left( 1.4276\times Hb+7.7 \right)\times\left( pH-7.4 \right) \right]$$

3. PCO_2_ from pH and BE

$$PCO_{2}=\frac{\left[ \frac{BE}{1-0.143\times Hb}-\left( 1.428\times Hb+7.7 \right)\times\left( pH-7.4 \right)+24.4 \right]}{0.03\times{10}^{pH-6.1}}$$

4. P50act (actual P50 is PO_2_ in mm Hg at 50% hemoglobin-oxygen saturation)

$$P50act=P50st\times{10}^{-0.48\times\left( pH-7.4 \right)}$$

P50st is standard P50 (the P50 at pH = 7.4 and PCO_2_ = 40 mm Hg).

1. Fractional saturation of hemoglobin (S) [5]

$$logitS=1.87+LN\left( \frac{PO_{2}}{1.955\times P50act} \right)+3.5\times TanH\left[ \frac{LN\frac{{PO}_{2}}{1.955\times P50act}}{3.5} \right]$$

$$S=\frac{EXP\left( logitS \right)}{1+EXP\left( logitS \right)}$$

LN is the natural logarithm.

TanH is the hyperbolic tangent function.

EXP (x) represents e raised to the power x.

1. BEcorr (Haldane correction in mEq/L)

$$\Delta S=S\left( initial \right)-S(final)$$

$$\Delta BE=0.22\times Hb\times\Delta S$$

$$BEcorr=BE+\Delta BE$$

2. VenAd (Venous admixture as %)

$$PiO_{2}=FiO_{2}\times\left( BP-47 \right)$$

$$PAO_{2}=PiO_{2}-\frac{Pa{CO}_{2}}{R}$$

$$CcO_{2}=1.34.Hb\times SAO_{2}+0.003\times P{AO}_{2}$$

$$CvO_{2}=1.34.Hb\times SvO_{2}+0.003\times P{vO}_{2}$$

$$CaO_{2}=1.34.Hb\times SaO_{2}+0.003\times P{aO}_{2}$$

$$VenAd=100\times\frac{CcO_{2}-CaO_{2}}{CcO_{2}-CvO_{2}}$$

$\mathrm{Pi}O_{2}=$ Inspired partial pressure of oxygen (mm Hg)

FiO_2_ = Fractional inspired concentration of oxygen

BP = Barometric pressure (mm Hg)

PAO_2_ = Partial pressure of oxygen in alveoli (mm Hg) as per alveolar gas equation

PaCO_2_ = Partial pressure of carbon dioxide in arterial blood (mm Hg)

R = Respiratory quotient

CcO_2_ = Oxygen content of “ideal” pulmonary capillary after equilibration (ml/dL)

CvO_2_ = Oxygen content of mixed venous blood (ml/dL)

CaO_2_ = Oxygen content of arterial blood (ml/dL)

SAO_2_ = Fractional saturation of hemoglobin with oxygen in “ideal” pulmonary capillary blood after equilibration

SvO_2_ = Fractional saturation of hemoglobin with oxygen in mixed venous blood

SaO_2_ = Fractional saturation of hemoglobin with oxygen in arterial blood

1. DO_2_ (Oxygen delivery in ml O_2_/min)

$$DO_{2}=CaO_{2}\times CO\times10$$

CO = Cardiac Output (L/min)

1. CvO_2_ (mixed venous oxygen content in ml O_2_/dL)

$$CvO_{2}=\frac{DO_{2}-VO_{2}}{10\times CO}$$

VO_2_ = Oxygen consumption (ml O_2_/min)

1. CvCO_2_ (Mixed venous CO_2_ content in ml CO_2_/dL)

$$CvCO_{2}=CaCO_{2}+0.1\times\frac{VCO_{2}}{CO}$$

CaCO_2_ = Arterial CO_2_ content (ml CO_2_/dL)

VCO_2_ = Carbon Dioxide expired (ml CO_2_/min)

1. PiO_2_ (Inspired partial pressure of O_2_ in mm Hg)

$$PiO_{2}=\left( BP-47 \right)\times FiO_{2}$$

2. PiN_2_ (Inspired partial pressure of N_2_ in mm Hg)

$$PiN_{2}=BP-47-PiO_{2}$$

3. CvN_2_ (Mixed venous N_2_ content - a simplifying approximation in ml N_2_/dL)

$$CvN_{2}=0.0017\times PiN_{2}$$

Lung unit equilibration targets

Let Q be the blood flow to the lung unit in ml.

1. V (Inspired gas volume in ml)

$$V=\frac{v}{q}\times Q$$

v/q = Ventilation perfusion ratio for the lung unit

1. ViO_2_ (Inspired O_2_ content in ml)

$${ViO}_{2}=V\times\frac{PiO_{2}}{BP}$$

2. O_2_ target (Lung unit total O_2_ content in ml)

$$O_{2} target={(CvO}_{2} x Q/10)+ViO_{2}$$

3. CO_2_ target (Lung unit total CO_2_ content in ml)

$$CO_{2} target=CvCO_{2} x Q/10$$

4. ViN_2_ (Inspired nitrogen content in ml)

$$ViN_{2}=V\times\frac{PiN_{2}}{BP}$$

5. N_2_ target (Lung unit total N_2_ content in ml)

$$N_{2} target=(CvN_{2} x Q/10)+ViN_{2}$$

Lung unit content calculations

1. CcapO_2_ (Alveolar capillary O_2_ content in ml/dL for the lung unit)

$$CcapO_{2}=1.34 x Hb\times ScapO_{2}+0.003\times PcapO_{2}$$

PcapO_2_ = lung unit capillary partial pressure of oxygen after equilibration in mm Hg

ScapO_2_ = lung unit capillary saturation of hemoglobin with oxygen after equilibration as a fraction

1. CalvO_2_ (Alveolar gas O_2_ content of lung unit in ml O_2_)

$$CalvO_{2}=Vexp\times\frac{PcapO_{2}}{BP}$$

Vexp = volume expired from the lung unit (ml)

1. Alveolar capillary CO_2_ content (CcapCO_2_) in ml CO_2_. Calculate by inserting PcapCO_2_ in Equations 2 and 3 above.

PcapCO_2_ = partial pressure of CO_2_ in alveolar capillary of lung unit after equilibration in mm Hg

1. CalvCO_2_ (Alveolar gas CO_2_ content of lung unit in ml CO_2_)

$$Calv{CO}_{2}=Vexp\times\frac{Pcap{CO}_{2}}{BP}$$

2. CcapN_2_ (Alveolar capillary N_2_ content post equilibration in ml N_2_)

$$CcapN_{2}=(BP-47-PcapO_{2}-Pcap{CO}_{2})\times0.0017$$

3. CalvN_2_ (Alveolar gas N_2_ content of lung unit in ml N_2_)

$$CalvN_{2}=Vexp\times\frac{(BP-47-PcapO_{2}-PcapCO_{2})}{BP}$$

4. CalvH_2_O (Alveolar gas H_2_O content in mm Hg)

$$CalvH_{2}O=Vexp\times\frac{47}{BP}$$

5. Vexp (Alveolar gas volume in ml)

$$Vexp=CalvO_{2}+CalvCO_{2}+CalvN_{2}+CalvH_{2}O$$

Equilibration by Mass Conservation (also see below ‘Embedding iterative calculations’)

1. Post equilibration PO_2_

$$CcapO_{2}+CalvO_{2}=O_{2}target$$

(resolved by iteration of PO_2_)

1. Post equilibration PCO_2_

$$CcapCO_{2}+CalvCO_{2}=CO_{2}target$$

(resolved by iteration of PCO_2_)

1. Post equilibration PN_2_

$$CcapN_{2}+CalvN_{2}=N_{2}target$$

(resolved by iteration of PO_2_ and PCO_2_)

Confirmation of compartmental equilibration

Equilibration is confirmed if $Blood R=Gas R$

1. Blood R calculation

$$Blood R=\frac{Cv{CO}_{2}-Ccap{CO}_{2}}{CcapO_{2}-CvO_{2}}$$

2. Gas R calculation

Calculation of mean PACO_2_

1. Vexptot (Total expiratory gas volume in ml)

$$Vexptot=\sum_{1}^{20} Vexp$$

2. mean PACO_2_ (Mean alveolar PCO_2_ in mm Hg)

$$mean PACO_{2}=BP\times\frac{VCO_{2}}{Vexptot}$$

Embedding iterative calculations

Several iterative calculations are embedded in the model. A useful feature of Excel software is that embedded iterations undergo automatic recalculation with every new input. An illustrative example is set out as follows:

1. A target value is set for a model calculation, for which the model estimate = f(operator).
2. For example, ‘O2 target’ (Equation 19) is the total oxygen content of a compartment calculated from the oxygen volumes in inspired gas and in mixed venous inflow. This value remains a fixed target during equilibration.
3. The model estimate of oxygen content for that compartment is $CcapO_{2}+CalvO_{2}$, and PO_2_ is the key operator.
4. The difference between the target and the model estimate is expressed as ‘difference = target - f(operator)’.
5. The File / Options / Formulas / Workbook Calculations / Enable Iterative Calculation box is checked, and the Maximum Iterations and Maximum Change are selected from dropdowns.
6. A statement which is invalid before equilibration is constructed using pre-equilibration model values. For example: Model value = Target + (0.01 x difference).
7. The statement becomes valid only when ‘difference = 0’, and the equilibration is therefore accomplished.
8. Excel software will iterate the operator value (in this case compartmental PO_2_) towards ‘difference = 0’ until the Maximum Iterations and / or Maximum Change selections are satisfied.

Back-calculations for the sensitivity analysis

This was a direct manipulation of the Adapted West model, not requiring ML. Once automatic re-calculations of BE and P50st and the iterative links were established ([Figure 3 (s)]), the nine inputs could be varied independently with immediate recalculation of shunt (plus log SD and mean) and VenAd.


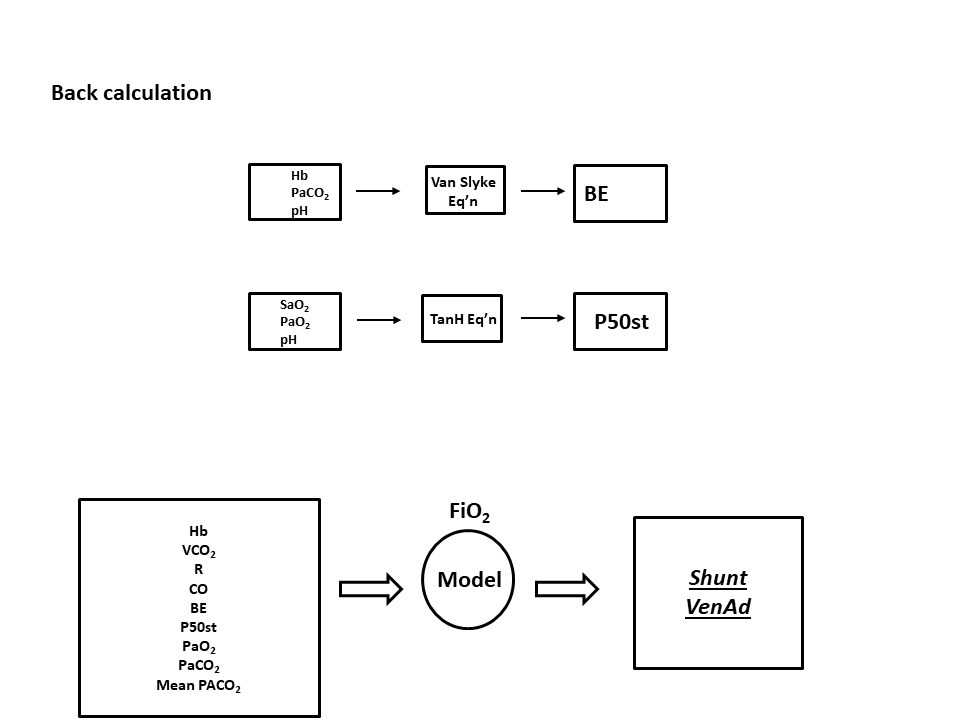


Figure 3 (s). Schematic of the back-calculation process for the sensitivity analysis. BE and P50st are model inputs which remain inherent in the blood gas data (Table 1). They were recalculated by re-application of Siggaard-Andersen’s Van Slyke and ‘tan H’ equations respectively (Equations 5 and 8).

The three key model descriptors shunt, log SD, and mean V/Q could then be recovered by iterative resolution of the following three ‘circular’ statements:

$$Mean \frac{V}{Q}=Mean \frac{V}{Q}+0.001\times(model PaCO_{2}-^{'}bedside^{'}PaCO_{2})$$

$$Log SD=Log SD+0.0001\times(model PaO_{2}-^{'}bedside^{'}PaO_{2})$$

$$Shunt=Shunt+0.001\times(model mean PACO_{2}-^{'}bedside^{'} mean PACO_{2})$$

The three statements are valid only when iterations of mean V/Q, log SD and shunt achieve concurrent equalization of model PaCO_2_, PaO_2_ and mean PACO_2_ values with their target ‘bedside’ values.

Corresponding venous admixture (VenAd) values are calculated via Equation 10.

Machine (Deep) Learning terminology [10, 11]

Keras model: A deep learning application programming interface designed to facilitate easier generation of complex neural models whilst retaining computational functionality.

ReLU: Rectified Linear Activation Unit. A function designed as an activator for training deep neural networks. The function returns the input value if that value is positive and zero if the value is negative.

RMSprop: Root Mean Square propagation. A method of adapting the learning rate for greater speed and efficiency.

Deep Learning Model - more detail [10-12]

The deep learning model (“the model”) consists of densely connected layers that transform the values of the features in the input layer to a single value in the output layer that is the model`s prediction of shunt. To illustrate the concept, Fig 4 (s) has a schema of a simpler model with three features in the input layer, four hidden layers, each consisting of seven nodes, and a single value in the output layer. By comparison, the model in this investigation has 10 features in the input layer and 6 hidden layers each with 128 nodes per layer.


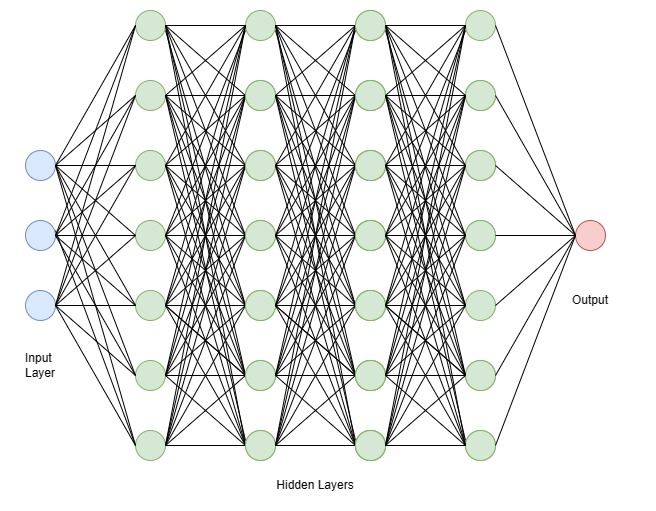


Figure 4 (s): Simplified schema of a small densely connected deep learning model

Each line represents a process by which the value at the node to the left is multiplied by a “weight” and undergoes addition with a “bias” value. The resultant inputs to each node on the right are summed and then subjected to an “activation function” to generate the value for that node. Principles of linear algebra are applied to facilitate vector and matrix multiplication and addition. The input layer, a vector of 10 features, is multiplied by a matrix containing 10 x 128 weights, yielding a vector of 128 values. This is added to a vector of 128 bias values. The result is then subjected to an activation function (“relu”) which returns the input value if the input is positive and zero if the input is negative. To form the second hidden layer, the first hidden layer (a vector) is multiplied by a 128 x 128 weight matrix, yielding a vector with 128 values to which is added another bias vector with 128 values and then subjected to an activation function yielding the second hidden layer. Thus, 128 x 10 weight variables and 128 bias variables (1408 variables) are needed to create the first hidden layer and 128 x 128 weight variables plus 128 bias variables (16512 variables) are needed to create each subsequent hidden layer. For the final output layer, the vector was multiplied by a 128-value vector to yield a single value to which was added a single bias value (thus, 129 variables) to yield the final result.

Each of the weight variables and bias variables are referred to as trainable parameters. Table 1 (s) is a summary of the model showing the trainable parameters applicable to each layer and the total of 84,097 trainable parameters for the model. Notably the activation function is nonlinear, otherwise the hidden layers could be collapsed to just one layer.

Table 1 (s): Keras - generated summary of the model. The first hidden layer is called “dense” and the second hidden layer “dense_1” and so on. The output shape “None” substitutes for batch size.


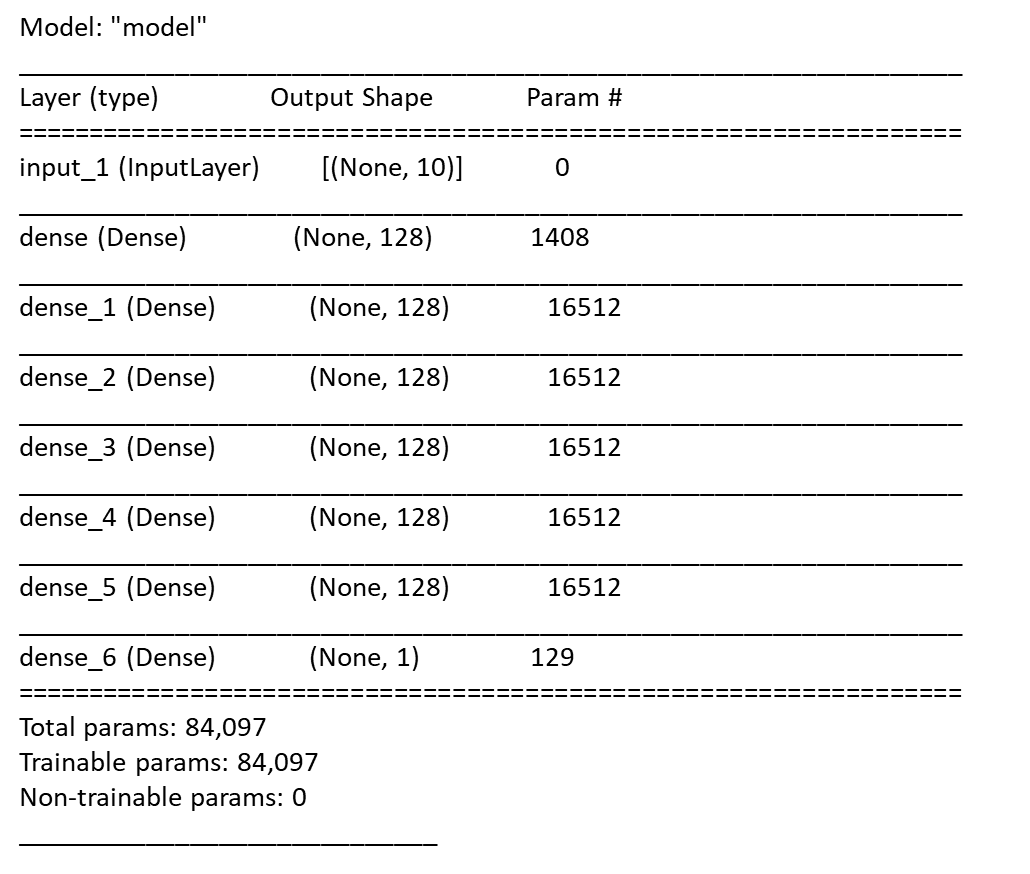


These 84,097 trainable parameters are initially assigned random values. The process of “training” the model involves adjusting these parameters so the model can predict shunt from previously unseen sets of features. Each sample of 10 features is transformed by the model to yield a predicted result. This is compared with the true result, and a “loss” function (the square of the error) is calculated. The average of loss functions for a batch of 256 samples (the “mean squared error”) is used by an “optimizer” algorithm (“RMSprop”) that incorporates a low learning rate, to work backwards to adjust the weights and bias variables with the aim of gradually minimizing the loss function. The next batch is then run forward through the model and another adjustment applied. “One epoch” refers to the whole dataset having been run through the model. Training continues with repeated epochs until satisfactory predictions are achieved.

The optimal number of epochs to train the model can be determined with validation curves which plot loss or another metric against the number of epochs. It is expected the model will gradually improve and eventually “overfit” the data which means that while the model can predict very well for the dataset on which it was trained, it will perform more poorly on unseen data. However, while the model requires tuning, it is best practice to not use the “test” data to tune the model. Rather the model should be fully tuned before any exposure to the test data. Thus, 20% of the training data was set aside as a validation set. The model was trained on the remaining 80% of the training data and evaluated using the validation set after each batch. It was expected that performance on validation data would improve to an optimum, but as training continues, overfitting would occur and performance on the validation data would deteriorate.


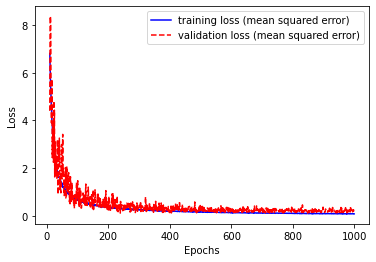


Figure 5 (s): Training and validation curves using loss


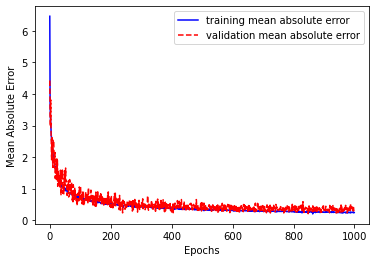


Figure 6 (s): Training and validation curves using the metric of mean absolute error

As can be seen from the validation curves in Figures 5 (s) and 6 (s), deterioration in performance did not occur in this investigation with ongoing training. The inability to induce overfitting likely reflects the mathematical derivation of the dataset and freedom from outliers due to observation errors.

As well as selecting the number of epochs for training, the validation curves were also used to tune other hyperparameters of the model including number of layers, number of nodes per layer and selection of the optimizer algorithm. The dataset was sufficiently large that the technique of k-fold validation was not required. Having been tuned, the model was subsequently trained on the full training dataset (including the validation set). The fully trained model was only then used to make predictions for the “test” dataset. Until this final step the model had not been exposed to the test dataset. Subsequently, no further tuning of the model occurred. The predictions on the test set were submitted as the results of the study for statistical analysis.

References

1. West JB, Wagner, PD. Pulmonary gas exchange. In: West JB, editor. Bioengineering Aspects of the Lung. New York: Marcel Dekker; 1977. p. 361-457.

2. Douglas AR, Jones NL, Reed JW. Calculation of whole blood CO2 content. J Appl Physiol (1985). 1988;65(1):473-7.

3. Kelman GR. Digital computer procedure for the conversion of PCO2 into blood CO2 content. Respir Physiol. 1967;3(1):111-5.

4. McHardy GJ. The relationship between the differences in pressure and content of carbon dioxide in arterial and venous blood. Clin Sci. 1967;32(2):299-309.

5. Siggaard-Andersen O, Siggaard-Andersen M, Fogh-Andersen N. The TANH-equation modified for the hemoglobin, oxygen, and carbon monoxide equilibrium. Scand J Clin Lab Invest Suppl. 1993;214:113-9.

6. Kelman GR. Digital computer subroutine for the conversion of oxygen tension into saturation. J Appl Physiol. 1966;21(4):1375-6.

7. Stainsby WN, Eitzman PD. Roles of CO2, O2, and acid in arteriovenous [H+] difference during muscle contractions. J Appl Physiol. 1988;65(4):1803-10.

8. West JB. Ventilation-perfusion inequality and overall gas exchange in computer models of the lung. Respir Physiol. 1969;7(1):88-110.

9. Siggaard-Andersen O. The Van Slyke equation. Scand J Clin Lab Invest Suppl. 1977;37(146):15-20.

10. Chollet F. Deep Learning with Python. Shelter Island, New York: Manning Publications Co; 2021.

11. Keras: The deep learning API. [Available from: <https://keras.io/>.

12. Raschka SM, V. . Python Machine Learning. 3rd ed. Birmingham: Packt Publishing Ltd; 2019.
